# Treatment of the Bleaching Effluent from Sulfite Pulp Production by Ceramic Membrane Filtration

**DOI:** 10.3390/membranes6010007

**Published:** 2015-12-31

**Authors:** Mehrdad Ebrahimi, Nadine Busse, Steffen Kerker, Oliver Schmitz, Markus Hilpert, Peter Czermak

**Affiliations:** 1 Institute of Bioprocess Engineering and Membrane Technology, University of Applied Sciences Mittelhessen, 35390 Giessen, Germany; mehrdad.ebrahimi@kmub.thm.de (M.E.); nadine.busse@kmub.thm.de (N.B.); steffen.kerker@kmub.thm.de (S.K.); Oliver.schmitz@kmub.thm.de (O.S.); 2 Sappi Fine Paper Europe, 89584 Ehingen, Germany; markus.hilpert@sappi.com; 3 Department of Chemical Engineering, Kansas State University, Manhattan, KS 66506, USA

**Keywords:** ceramic membrane, bleaching effluents, waste water treatment, chemical oxygen demand removal, membrane fouling, permeate flux rate

## Abstract

Pulp and paper waste water is one of the major sources of industrial water pollution. This study tested the suitability of ceramic tubular membrane technology as an alternative to conventional waste water treatment in the pulp and paper industry. In this context, in series batch and semi-batch membrane processes comprising microfiltration, ultrafiltration and nanofiltration, ceramic membranes were developed to reduce the chemical oxygen demand (COD) and remove residual lignin from the effluent flow during sulfite pulp production. A comparison of the ceramic membranes in terms of separation efficiency and performance revealed that the two-stage process configuration with microfiltration followed by ultrafiltration was most suitable for the efficient treatment of the alkaline bleaching effluent tested herein, reducing the COD concentration and residual lignin levels by more than 35% and 70%, respectively.

## 1. Introduction

**Figure 1:**
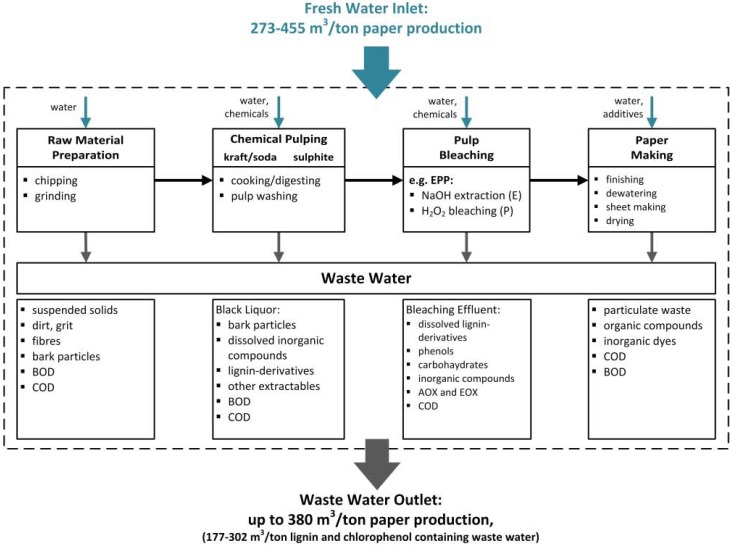
Schematic illustration of a paper production process (modified from [3,5,6,13,14]). AOX: absorbable organic halogens; BOD: biological oxygen demand; COD: chemical oxygen demand; EOX: extractable organic halogens

Waste water reuse is a globally imperative component of sustainable water management [1]. The pulp and paper industry produces substantial volumes of polluted waste water [2] (~220–380 m^3^ per ton paper [3]) and is also one of the largest consumers of fresh water (~273–455 m^3^ per ton paper [3]) [1,4,5] (Figure 1). The volume and characteristics of waste water differ according to the type of raw material, the process technologies (e.g., mechanical or chemical pulping, pulp bleaching), whether or not there is internal recirculation of the effluent [4,6], and the type of paper product [4] (Figure 1). Cellulosic pulp can be manufactured by chemical or mechanical pulping, but chemical pulping is the most prevalent technology [7]. Chemical pulping and bleaching generates enormous amounts of waste water containing heterogeneous mixtures of organic and inorganic compounds (including polydisperse lignin-derived polymers) that cause discoloration as well as high chemical oxygen demand (COD), biological oxygen demand (BOD) and toxicity [5]. Although efforts to achieve sustainable onsite water management have increased over the last few decades [8,9], there is still room for improvement [2], and paper manufacturers must develop more efficient waste water strategies. Membrane filtration can be used as the basis of so-called “advanced treatment processes” which are inexpensive and have a small footprint, resulting in high quality effluents [1].

Membrane filtration is already established in the pulp and paper industry to achieve the efficient recovery of waste materials, impurities and by-products from effluent prior to discharge. Membrane filtration can also be used to concentrate and purify particular effluent components while saving space on the production line due to the high packing density of membrane plants. A variety of different membrane types, materials and geometries have been developed in the last few decades. The most important applications in the pulp and paper industry are the recovery of valuable products such as the fractionation and purification of lignosulfonate from spent sulfite liquor and the fractionation and purification of lignin from kraft black liquor. Membrane filtration also saves energy because it can concentrate dilute spent sulfite more efficiently than evaporation, and is environmentally beneficial because it allows the purification of kraft bleach effluent [38].

In most previous studies, polymeric and ceramic membranes have been used to reduce the COD and to separate lignin-derived compounds from kraft black liquors (Table 1). Waste water treatment is the largest application area for ceramic membranes, particularly in filtration processes where polymeric membranes are unsuitable. Polymeric membranes are lighter, less expensive, more robust and less prone to fouling [9], but ceramic membranes last longer, they are hydrophilic, they achieve higher permeate fluxes, and they offer greater thermal, chemical and mechanical stability, the latter being particularly valuable when filtering abrasive media with large pressure gradients [11].

**Table 1:** Overview of recent studies investigating membrane technologies for the treatment of wastewater from pulp and paper mills.

Process	Source	Membrane type	Reference
RO	Kraft black liquor	Spiral-wound	[22]
RO	Bleaching effluent	Spiral-wound	[34]
NF	Kraft black liquor	Tubular ceramic membranes	[15,21,35]
NF	Bleaching effluent	Spiral-wound	[34]
NF	Kraft black liquor	Tubular polymeric membranes	[15,22]
UF/NF	Hardwood black liquor	Tubular ceramic membranes	[28]
UF	Kraft black liquor	Rotating disc module using cellulose triacetate	[32]
UF	Kraft black liquor	Tubular polymeric membranes	[18,25]
UF	Kraft black liquor	Polymeric flat membranes	[33]
UF	Bleaching effluent	Spiral-wound	[34]
UF	Cooking liquor	Tubular ceramic membranes	[23,25]
UF	Kraft black liquor	Tubular ceramic membranes	[15,16,19,20,21,23,24,25,27,28,29,30,35]
UF	Kraft black liquor	Tubular polymeric membranes	[18,25]
UF	Acidic white water, acidic clear filtrate	Cross rotational filter	[17]
MF		Tubular polymeric	[17]
MF	Kraft black liquor	Polymeric flat membranes	[33]
MF	Kraft black liquor	Tubular ceramic membranes	[16,22,31,33]

Bleached sulfite pulp mills produce large quantities of brown-colored effluents with high COD, high levels of lignin and their partially aromatic degradation products, and polymeric materials that resist biological degradation. These effluents must be treated to remove particulate residual lignin and COD prior to biological treatment. This investigation considered the processing of alkaline bleaching effluent from sulfite pulp production by successive fractionation using tubular ceramic membranes, aiming to minimize the COD and thus reduce waste disposal costs while recovering valuable waste products and by-products such as lignin derivatives (e.g., lignosulfonates). Filtration experiments were carried out in batch and semi-batch mode, focusing on the influence of the most important process parameters such as ceramic membrane pore size, transmembrane pressure (TMP) and crossflow velocity (CFV) on the permeate flux behavior and separation/retention characteristics. The efficient recovery of lignin derivatives (also known as technical/industrial lignin) is beneficial and industrially relevant because these highly aromatic molecules can be converted into biobased products such as biofuels, polymer modifiers, resins, binders and fine chemicals like vanillin, quinones, phenols or benzene/toluene/xylene (BTX) products [12].

## 2. Materials and Methods

### 2.1. Materials

**Figure 2:**
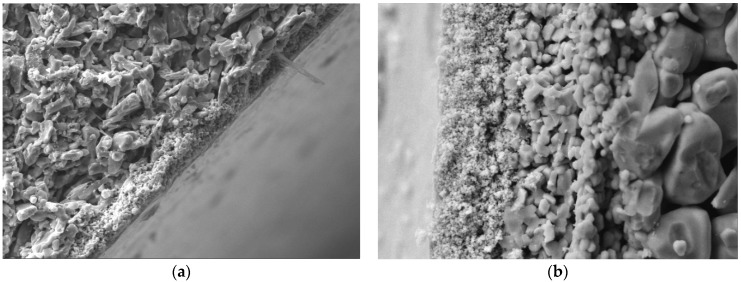
Cross-sectional scanning electron micrograph of the two ceramic membranes used in this investigation: 20 kDa (**a**) and a 1 kDa (**b**).

The ceramic membranes used in this investigation were asymmetric in structure, consisting of one support layer with large pores and a low pressure drop and one or more separation layers to control the permeation flux (Figure 2). One separation layer was suitable for microfiltration (MF) whereas two or more were required for ultrafiltration (UF) and nanofiltration (NF). The characteristics of the different ceramic membranes are summarized in Table 2. The alkaline bleach effluent tested herein was supplied by Sappi Fine Paper Europe, Ehingen Mill, Germany. The properties of the effluent are summarized in Table 3.

**Table 2:** Characteristics of the ceramic membranes used in this investigation.

Membrane	MF	UF	NF	NF
Material	Al_2_O_3_/Al_2_O_3_	Al_2_O_3_/TiO_2_	TiO_2_	TiO_2_/TiO_2_
Cutoff	0.1 µm, 0.14 µm, 0.2 µm	5 kDa, 20 kDa, 0.05 µm	1000 Da	1000 Da
pH	0–14	0–14	0–14	0–14
Temp. Max.	121 °C	121 °C	150 °C	121 °C

**Table 3:** Characteristics of the alkaline bleach effluent used in this investigation.

Parameter	Unit	Bleaching effluent	Variation Range
COD	mg·L^−1^	10,400	10,300–12,000
pH value		10.65	10.0–11.0
Temperature	°C	60	60–70
Conductivity	mS·cm^−1^	8.0	8.0–10
Viscosity	pas	6.0	5.85–6.08
TOC	mg·L^−1^	4000	3500–4500
Na	mg·L^−1^	2430	1800–3300

### 2.2. Water Quality Assessment

Waste water analysis was applied to permeate and retentate samples. The COD, BOD and total organic carbon (TOC) were determined using Photometer Photolab S6 (Wissenschaftlich–Technische Werkstätten GmbH, Weilheim, Germany). The conductivity was measured using a multi-range conductivity meter (HI 9033, Hanna Instruments, Kehl am Rhein, Germany) and the pH was determined using a digital potentiometer. Lignin levels were determined by measuring absorbance at 280 nm using a Helios Gamma UV/VIS spectrophotometer. The efficiency of lignin removal was calculated by comparing the concentrations of lignin in the feed solution and permeate.

### 2.3. Experimental Setup

**Figure 3:**
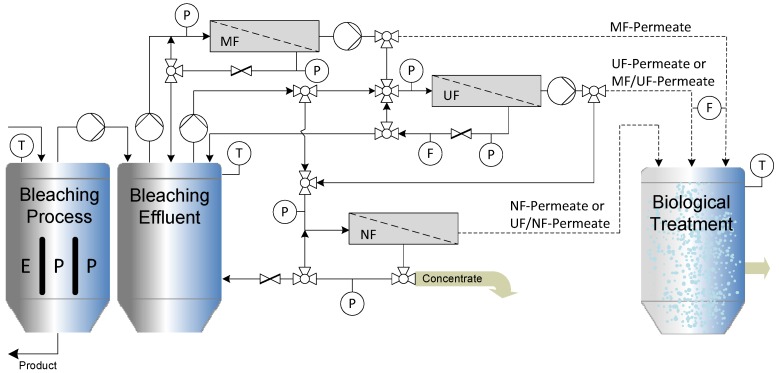
Schematic illustration of the laboratory-scale multi-stage cross-flow filtration system.

The setup of the multi-stage cross-flow ceramic membrane filtration process is presented schematically in Figure 3. Cross-flow filtration (MF, UF and NF) of the alkaline bleaching effluent was achieved using two stirred batch-reactor systems with the membrane modules arranged in parallel. In order to optimize the treatment process, various commercial ceramic membranes with different molecular weight cut-off (MWCO) values (Table 2) were evaluated by measuring the permeate flux rate and COD in the permeate and retentate.

The percentage efficiency of COD reduction at time *t* was calculated by comparing the concentrations of COD in the permeate samples with those in the concentrate samples as follows:

(1) $$R_{t}=\left(1-\frac{C_{p\left(t\right)}}{C_{c\left(t\right)}}\right)\times 100\%$$

where $R_{t}$ is the percentage efficiency of COD reduction at time *t*, $C_{p\left(t\right)}$ is the COD concentration in the permeate samples in mg·L^−1^ at time *t*, and $C_{c\left(t\right)}$ is the COD concentration in the concentrate in mg·L^−1^ at the same time. The operating conditions during MF, UF and NF involved different TMPs of 1–5 bar and a constant temperature of 60 °C. TMP is the driving force for membrane separations and is defined as the difference between the retentate and permeate stream pressures:

(2) $$TMP=\left(\frac{P_{1}+\;P_{2}}{2}\right)-P_{3}$$

where *TMP* is the total transmembrane pressure, *P*_1_ is the inlet pressure, *P*_2_ is the retentate line pressure and *P*_3_ is the negative permeate pressure. The permeate flow rate was measured using a digital flowmeter.

### 2.4. Membrane Cleaning

Before and after each filtration run, the membranes were cleaned for at least 1 h with 1% (w/v) NaOH at 60 °C and rinsed with distilled water to remove any compounds remaining in the system. In this study, the chemical cleaning efficiency of fouled ceramic membranes was evaluated by comparing the initial clean water flux before filtration and after each chemical cleaning process as follows:

(3) $$Chemical\;cleaning\;effectivity=\left(\frac{Clean\;water\;flux\;after\;cleaning}{Clean\;water\;flux\;of\;unused\;membrane}\right)\times \;100\%$$

## 3. Results and Discussion

### 3.1. Multi-Stage Separation Membrane Processes

**Figure 4:**
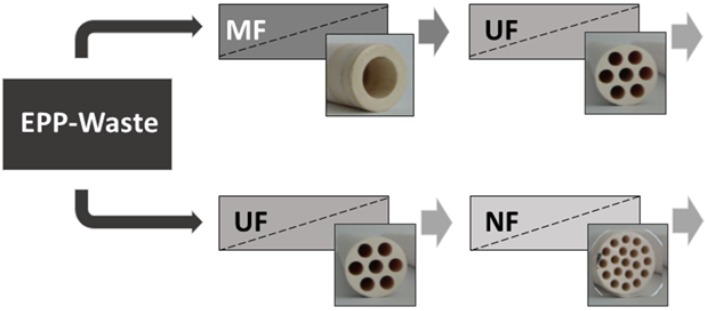
Overview of the two-stage membrane filtration configurations in this investigation.

Two different multi-stage membrane processes based on ceramic tubular membranes were investigated: MF➔UF and UF➔NF. These processes are summarized in Figure 4.

The main objective was to identify the membrane pore sizes that optimize separation efficiency, the number of treatment stages required and the optimal operating conditions for the efficient treatment of alkaline bleaching effluent. All of the membrane processes were characterized in terms of flux behavior, the efficiency of COD removal, chemical cleaning and back flushing, and the recoverable permeate fluxes. For the evaluation of chemical cleaning and back flushing efficiency, clean water tests were carried out at different temperatures and TMPs to determine and compare membrane productivity before and after each filtration experiment.

### 3.2. Two-Stage Membrane Filtration Process (MF➔UF)

A two-stage (MF➔UF) ceramic membrane process was developed for the efficient removal of COD from alkaline bleaching effluent. The optimization of operating conditions is important for the efficiency of a membrane filtration system, so the available ceramic membranes were screened and the most appropriate membranes were pre-selected. Two operating parameters (CFV and TMP) were varied, and samples from the permeate and retentate streams were collected for analysis. The retention rates of COD and lignin from representative microfiltration experiments in semi-batch mode are reported in Table 4. All filtration experiments were conducted in triplicate and the results are presented as experimental means. The experiments were carried out using ceramic MF membranes with different pore sizes (0.1 0.14 and 0.2 µm) at a constant temperature of 60 °C, a low CFV (0.15–0. 51 m·s^−1^) and a TMP of 1 or 2 bar.

**Table 4:** Selected performance data for 0.1, 0.14 and 0.2 µm ceramic microfiltration membranes (n. a. = not available).

Membrane		TMP	CFV	Re	COD Removal	Lignin Removal
Cut-off	Channel configuration	(bar)	(m·s^−1^)		(%)	(%)
0.1 µm	Mono-channel	2.0	0.25	9549	27.81	20.0
0.1 µm	Mono-channel	1.0	0.37	8639	34.09	n. a.
0.1 µm	Mono-channel	2.0	5.6	150,000	28.04	42.1
0.1 µm	Seven-channel	1.0	0.26	16,370	21.28	31.0
0.14 µm	Eight-channel	2.0	0.15	15,302	37.96	n. a.
0.2 µm	Seven-channel	2.0	0.51	34,210	23.16	29.2
0.2 µm	Nineteen-channel	2.0	0.26	19,108	19.65	28.0
0.2 µm	Mono-channel	2.0	0.24	9549	32.83	n. a.
0.2 µm	Mono-channel	2.0	0.27	9549	37.51	n. a.
0.2 µm	Mono-channel	2.0	0.29	9549	32.81	n. a.

**Figure 5:**
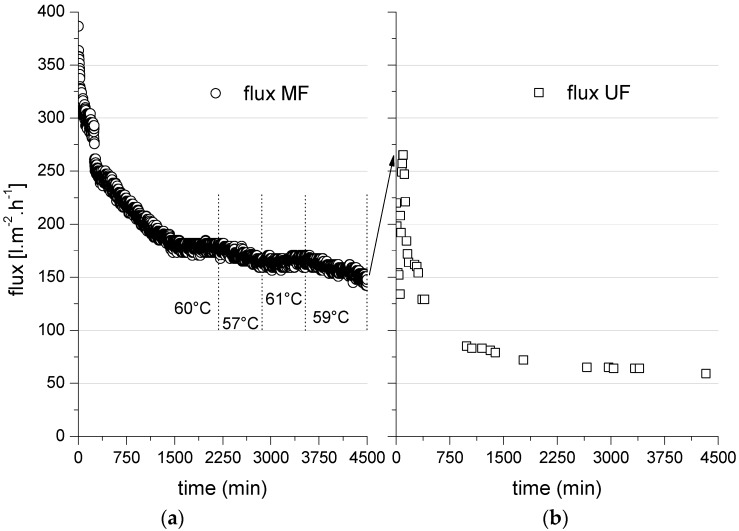
Permeate flux *versus* time for a 0.1-µm MF ceramic membrane (**a**) as a first treatment step (feed = bleaching effluent) followed by a 20-kDa UF membrane (**b**) as second treatment step (feed = MF permeate). TMP = 2 bar; temperature = 60 °C except where shown otherwise.

Figure 5 shows a representative permeate flux *versus* time analysis for the treatment of bleaching effluent using a 0.1-µm MF ceramic membrane followed by a 20-kDa UF membrane with the MF permeate as the feed. In this two-stage filtration experiment, the average flux values for the MF and UF membranes were 186 and 140 L·m^−2^·h^−1^, respectively, for a duration of 75 h and a TMP of 2 bar.

**Figure 6:**
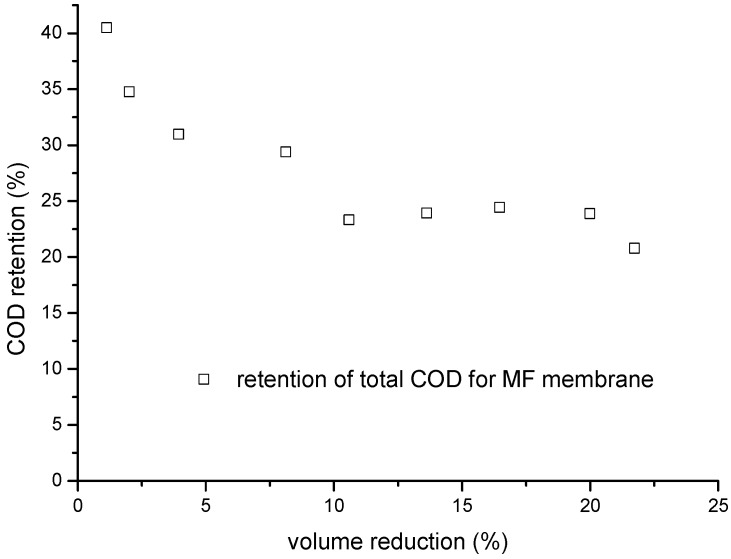
Effect of volume reduction on the COD retention efficiency of a ceramic 0.1-µm MF membrane.

The total COD changed as the volume of the MF process was reduced (Figure 6). In this MF experiment, a flux of 301 L·m^−2^·h^−1^ was achieved at a volume reduction of ~35% over 2 h, and a flux of 145 L·m^−2^·h^−1^ was achieved at a volume reduction of 21% over 80 h. The initial retention efficiency of COD was ~40%, but this started to increase at a volume reduction of up to 2%.

The MF membrane flux was strongly dependent on the temperature of the feed stream. The left panel of Figure 5 shows that even a minimal change in the feed temperature had a substantial effect on the performance of the ceramic membrane. Varying the feed temperature between 57 and 61 °C affected the membrane flux due to the impact of temperature on the feed viscosity. The permeate flux across the UF membrane fell from its initial value of 220 to 59 L·m^−2^·h^−1^ over an operating time of 75 h at 2 bar TMP and a cross-flow velocity of 4 m·s^−1^ due to membrane fouling by the MF permeate. The 0.1-µm MF membrane and 20-kDa UF membrane reduced the COD in the permeate by 30% and 25%, respectively, resulting in an overall reduction of 35%–40% during the two-stage process (Table 5). Many previous investigations of ceramic membrane processes have considered the concentration of COD and the recovery of lignin or lignin fractions. However, nearly all previous reports have focused on the treatment and fractionation of lignin from kraft black liquors, which have a vastly different composition compared to the bleaching effluent discussed herein (Table 1). Wallberg and Jönssen [19] achieved comparable results when testing the influence of ceramic UF membrane cut-off values during the treatment of kraft black liquor, and they achieved a lignin removal efficiency of ~32% using a 15-kDa ceramic UF membrane.

All the ultrafiltration experiments were conducted in triplicate and the results are presented as the means. Table 6 shows the reproducibility of these data for a 20-kDa ceramic UF membrane with MF permeate as the feed solution.

**Table 5:** Performance data for 0.1-µm and 20-kDa ceramic membranes in series.

Membrane Process	CFV	Re	TMP	COD Removal	Lignin Removal	pH_t0_	pH_end_
MF, 0.1 μm	5.6 m·s^−1^	150,000	2.0 bar	28%–30%	40%–50%	11.2	10.1
UF, 20 kDa	4.0 m·s^−1^	41,000	2.0 bar	20%–30%	30%–40%	10.1	9.2
Total removal				35%–45%	60%–73%		

**Table 6:** Representative performance reproducibility data for 20-kDa ceramic membranes.

Membrane	TMP (bar)	CFV (m·s^−1^)	COD removal (%)
UF, 20 kDa	2.0	4.10	20.32
UF, 20 kDa	2.0	4.00	24.23
UF, 20 kDa	2.0	4.10	28.40
UF, 20 kDa	1.0	0.43	42.30
UF, 20 kDa	1.0	0.46	40.20
UF, 20 kDa	1.0	0.46	37.33

### 3.3. The Effect of Back Flushing on MF Performance

One of the most important processes affecting the performance and productivity of membrane filtration systems is fouling, which causes a dramatic increase in membrane resistance during filtration due to the formation of unwanted deposits on the membrane surface and/or pores [36]. It is therefore necessary to develop strategies that prevent or reduce membrane fouling to minimize its impact. Back flushing (an operational technique in which the TMP is periodically inverted) is widely used to clean the filters in industrial processes, and is often used to complement mechanical cleaning procedures. The main back flushing parameters are duration, frequency, back pressure and flux. A series of filtration experiments was therefore carried out to investigate the effect of different back flushing frequencies on the permeate flux behavior of a ceramic MF membrane, compared to an identical process without back flushing. All experiments were carried out with a constant back flush pressure of 4 bar and the same back flush duration of ~10 s.

**Figure 7:**
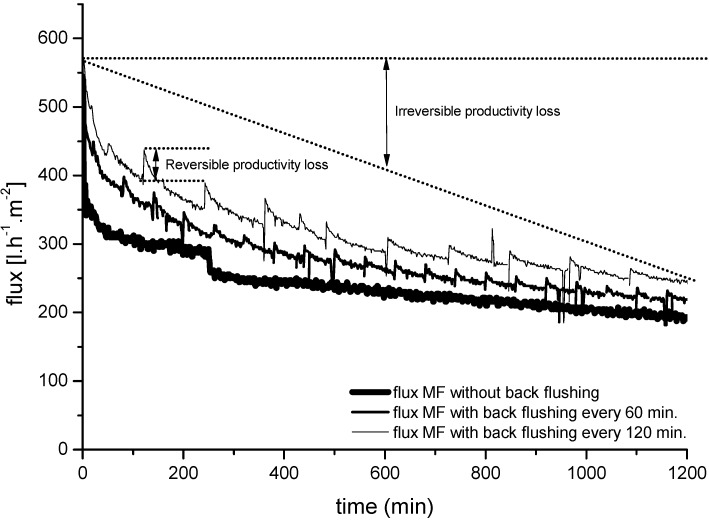
Influence of back flushing frequencies (60 and 120 min) on permeate flux through a 0.1-µm ceramic MF membrane (mono-channel design) during the filtration of bleaching effluents. TMP = 2 bar; temperature = 60 °C.

Figure 7 shows the effect of two different back flushing frequencies on the permeate flux through the ceramic MF membrane compared to the same type of membrane under the same operating conditions without back flushing. These tests were carried out using the experimental setup shown in the MF/UF/NF hybrid membrane system (Figure 3) without the UF/NF loop. The permeate flux values were plotted as a function of the duration of forward filtration (3 h) and back pulse frequencies of 60 and 120 min.

These results show that MF ceramic membranes respond well to back flushing and can maintain permeate flux at a high level for the duration of the experiment. Without back flushing, the average flux was 240 L·m^−2^·h^−1^. With a back flushing frequency of 60 min, the flux increased by up to 16% to an average flux of 279 L·m^−2^·h^−1^, and with a back flushing frequency of 120 min, the flux increased by up to 48% to an average flux of 314 L·m^−2^·h^−1^. The effect of back flushing was more pronounced when the back flush frequency was longer at a constant back pulse pressure.

### 3.4. Two-Stage Membrane Filtration Process (UF➔NF)

**Figure 8:**
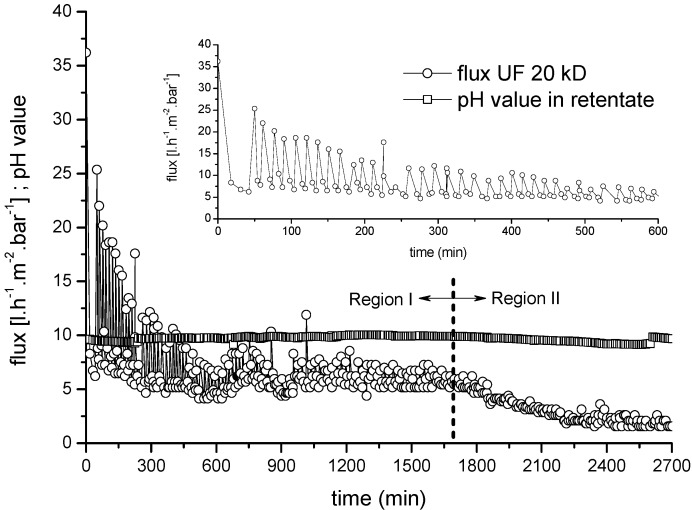
Normalized average flux rate and flux degradation for a 20-kDa ceramic UF membrane as a first treatment step (feed = bleaching effluent). CFV = 0.3 m·s^−1^; temperature = 60 °C; TMP = 2.0 bar.

The flux behavior of a second two-stage process (UF➔NF) was investigated using the same alkaline bleaching effluent. The UF stage comprised a 20-kDa ceramic membrane with a seven-channel configuration operated as a semi-batch process, a temperature of 60 °C, an average low CFV of 0.3 m·s^−1^ and a TMP of 2 bar. The decline in flux over time during the UF and NF processes are shown in Figure 8 and Figure 11, respectively. Again, membrane fouling was shown to reduce the productivity of filtration, causing the flux to decline over time under constant TMP. Figure 8 shows that the permeate flux through the UF membrane declined continuously from the initial value of 36.2 L·m^−2^·h^−1^·bar^−1^ to 5.1 L·m^−2^·h^−1^·bar^−1^ after 6 h and then to 2.0 L·m^−2^·h^−1^·bar^−1^ over the remaining 40 h. The flux behavior of the membrane over time could be divided into two broad phases. The first phase was characterized by a rapid drop in the permeate flux from 37 to ~5 L·m^−2^·h^−1^ during the first 45 min of filtration, whereupon a stable average permeate flux of 9 L·m^−2^·h^−1^ could be achieved by periodic back flushing. The second phase was characterized by a gradual further decline in membrane performance over the next 10 h from 9 to 2.5 L·m^−2^·h^−1^. The initial pH value in the retentate stream only changed marginally following UF for 45 h.

**Figure 9:**
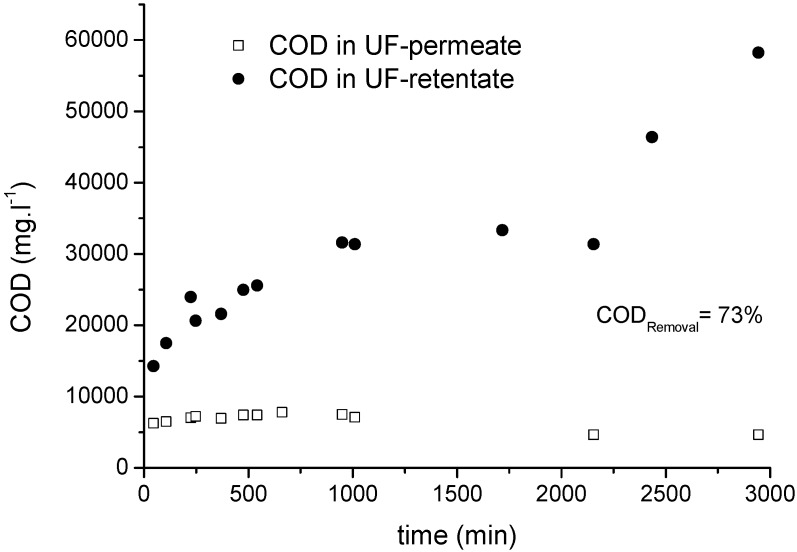
Change in COD concentration in permeate and retentate samples during an UF process (20-kDa membrane) using MF permeate as the feed.

Figure 9 shows the trend in COD concentrations in the permeate and retentate samples and UF process in semi batch mode for 50 h, resulting in a maximum COD removal efficiency of 73%. The COD in the retentate samples increased rapidly during the first 10 h of filtration due to the high initial permeate flux in this period. After stabilization of the permeate flux, the CSB increased almost linearly as typical for a fed-batch operation. A sudden increase in the retentate CSB near the end of the filtration reflects the termination of the feed supply as part of the batch operation.

### 3.5. The Effect of Back Flushing on UF Performance

**Figure 10:**
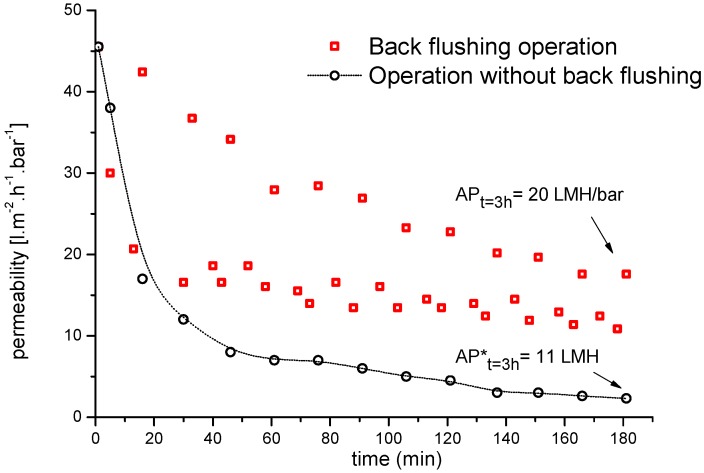
Average permeability of a 20-kDa ceramic UF membrane in semi-batch mode with and without back flushing during the filtration of bleaching effluent. Temperature = 60 °C; back flushing duration 2–10 s every 15 min at 4 bar (AP = average permeability).

Regardless of operational fouling control, regular membrane cleaning is needed to remove foulants and maintain permeability losses within a given interval [37]. Therefore, in most of the crossflow UF systems tested herein, back flushing was used to remove the fouling layer. The collected permeate was periodically pumped back through the membrane (out–in mode) using 2–3 bar air pressure for a duration of 4 s, every 20 min. Figure 10 shows the corresponding change in UF membrane flux with and without back flushing as a cleaning process, and their influence on the regeneration of membrane permeability during UF.

Compared to the uncleaned ceramic UF membrane without back flushing, the membrane cleaned by back flushing during a 3 h filtration run showed about a 45% increase in average membrane permeability (from 11 to 20 L·m^−2^·h^−1^). These results indicate that the membrane permeate flux increased when back flushing was applied regardless of the feed characteristics and process conditions. Another important outcome was that periodic back flushing did not negatively affect the permeate quality in terms of COD removal efficiency.

**Figure 11:**
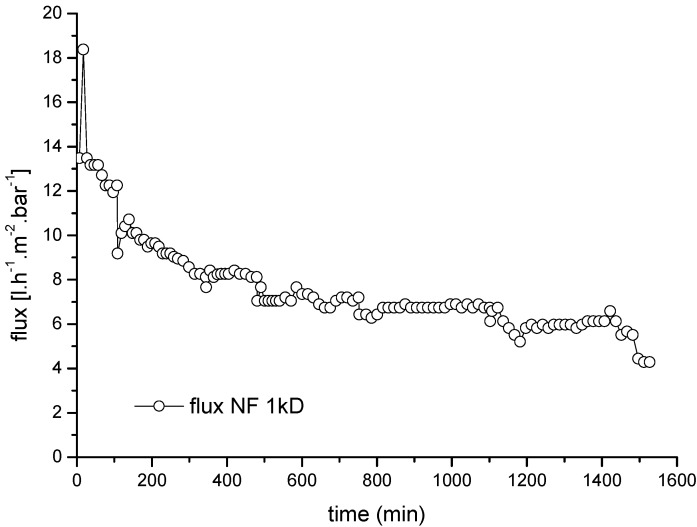
Normalized average flux rate and flux degradation for a 1-kDa ceramic NF membrane (second stage) with the permeate from an upstream UF membrane as the feed solution. CFV = 3.2 × 10^−5^ m·s^−1^; temperature = 60 °C; TMP = 2.0 bar.

Figure 11 shows a representative normalized flux–time curve for the ceramic NF membrane used as the second treatment stage, with the UF permeate as the feed. The average COD and lignin retention efficiencies achieved during the UF➔NF process were ~40% and ~66%, respectively (Table 7). Toledano *et al.* [30] investigated the separation and fractionation of lignin from black liquor using ceramic UF membranes with different cut-off values. They achieved lignin removal efficiencies of 19%, 34% and 81% for cut-off values of 15, 10 and 5 kDa, respectively.

**Table 7:** Performance data for 20-kDa and 1-kDa ceramic membranes in series.

Membrane Process	TMP	COD Removal	Lignin Removal	pH_t0_	pH_end_
UF, 20 kDa	2.0 bar	30%–35%	45%–50%	11.2	9.2
NF, 1 kDa	2.0 bar	20%–27%	30%–40%	9.2	8.2
Total removal		35%–40%	45%–66%		

### 3.6. Clean Water Flux and the Efficiency of Membrane Chemical Cleaning

**Figure 12:**
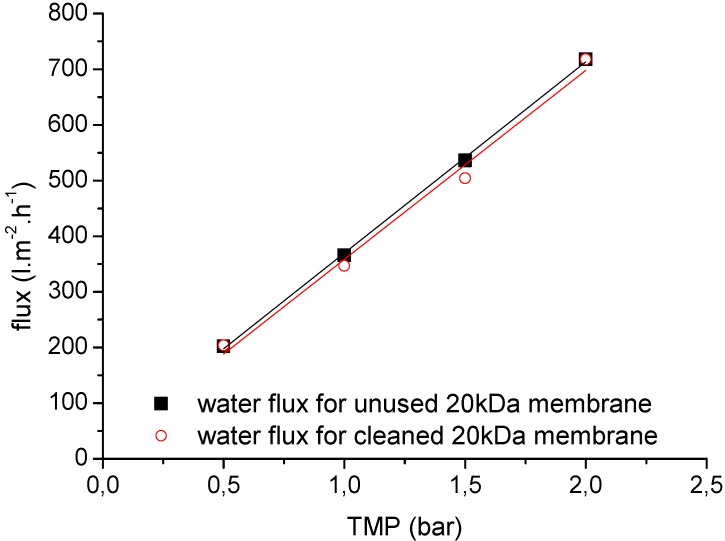
The clean water fluxes of unused and cleaned 20-kDa UF ceramic membranes at different TMPs (0.5–2.0 bar) and a temperature of 25 °C.

**Figure 13:**
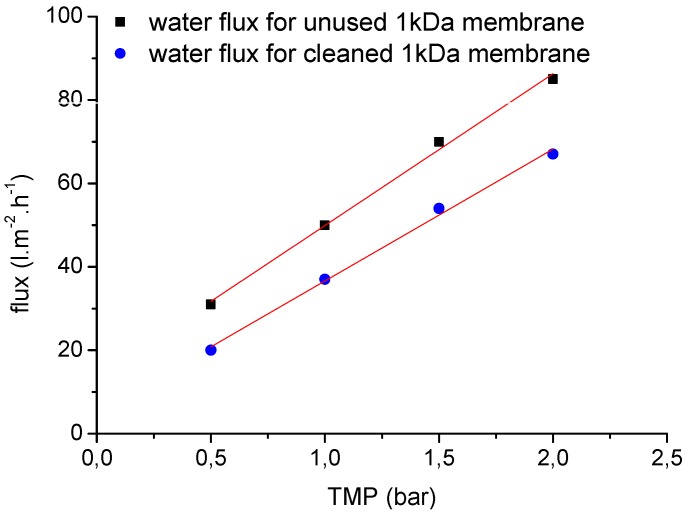
The clean water fluxes of unused and cleaned 1-kDa NF ceramic membranes at different TMPs (0.5–2.0 bar) and a temperature of 60 °C.

One of the most important concerns for the application of the membranes is fouling, and chemical cleaning is therefore an integral operation for membrane filtration systems during wastewater treatment. Systematic filtration experiments were therefore carried out to investigate the chemical cleaning efficiency of the selected ceramic membranes. Figure 12 and Figure 13 show representative clean water flux measurements before each filtration experiment and after chemical cleaning as a function of TMP (0.5, 1.0, 1.5 and 2.0 bar) and temperature (25 °C and 60 °C) for two ceramic membranes (20 kDa and 1 kDa). After membrane cleaning with alkaline agents, the water permeability of the 20-kDa membrane could be regenerated to ~98% of the performance of the unused membrane (Figure 12). Overall, an average chemical cleaning efficiency of 70%–80% was achieved for the 1-kDa ceramic membranes, depending on the process parameters and duration of filtration (Figure 13).

## 4. Conclusions

This investigation aimed to develop an efficient multistage ceramic membrane process for the treatment of bleaching effluents generated during sulfite pulp production. This effluent is highly polluted and must be treated prior to biological processing to achieve a noticeable reduction in the COD load so that the subsequent biological treatment remains efficient. It must also be treated before final disposal as a key strategy to improve environment protection and resource recovery. Finally, further treatment is required before water reuse to limit the volume of discharged waste water. Ceramic membrane technology can achieve a significant reduction in the level of organic matter, measured as the COD load and the lignin content.

To achieve the aims discussed above, the performance of single-stage and multi-stage cross-flow MF, UF and NF systems was tested in different configurations to determine the most efficient strategy for the reduction of COD and residual lignin in pulp mill bleaching effluents using ceramic tubular membranes. A comparison of the ceramic MF, UF and NF membranes in terms of separation efficiency and performance revealed that the two-stage process with the MF➔UF configuration was most suitable for the efficient treatment of the alkaline bleaching effluent tested herein, reducing the COD concentration by 45% and residual lignin levels by 73%.

Single MF ceramic membranes with different pore sizes and without feed pre-treatment achieved a 20%–40% reduction in the COD concentration and residual lignin levels, depending on the precise process conditions. Although a significant reduction in the COD concentration could also be achieved using a single UF membrane, the permeate flux was a limiting factor preventing the realization of an efficient single-step UF process. The susceptibility of UF membranes to fouling reduced their performance by up to 80%. However, UF membrane performance could be increased dramatically by placing MF and UF membranes in series in a two-step process so that the membrane permeability was stabilized after a certain period of filtration even without backwashing.

Cleaning strategies for the ceramic membranes were investigated to increase and/or stabilize membrane performance during filtration. Efficient chemical cleaning methods for fouled membranes were combined with back flushing to establish a successful cleaning process.

The use of ceramic tubular membranes for the treatment of alkaline bleaching effluent provides substantial economic and environmental benefits, as demonstrated by the reduction of COD concentrations and residual lignin levels achieved herein. When applied to industrial processes, this could accomplish a significant reduction in the volume of untreated bleaching effluent discharged from paper mills, thus substantially reducing their environmental impact.
